# Reactivity of Ruthenium Vinylidene Complexes Containing Indenyl/dppe Ligands and Unsaturated Bonds at Cδ with Trimethylsilyl Azide

**DOI:** 10.3390/molecules17078533

**Published:** 2012-07-17

**Authors:** Hui-Ling Sung, Tze-Min Her, Wen-Hsien Su, Chin-Pao Cheng

**Affiliations:** 1 Department of Mathematics and Science (Pre-college), National Taiwan Normal University, New Taipei City 244, Taiwan; 2 Department of Chemical and Materials Engineering, Lunghwa University of Science and Technology, Taoyuan County 333, Taiwan; Email: ch007@mail.lhu.edu.tw (T.-M.H.); cower1987.tw@hotmail.com (W.-H.S.); 3 Department of Mechatronic Technology, National Taiwan Normal University, Taipei 106, Taiwan; Email: cpcheng@ntnu.edu.tw

**Keywords:** vinylidene, ruthenium, *N*-coordinated, cyclopropenyl, furyl, indenyl

## Abstract

This study presents a new reaction of cationic vinylidene complexes with Me_3_SiN_3_ (TMSN_3_), which yields *N*-coordinated nitrile complexes **3**. Treatment of a ruthenium acetylide precursor containing indenyl and dppe ligands with a series of organic halides produced the corresponding vinylidene complexes **2** in good yield. Further reaction of **2** with TMSN_3_ at room temperature produced *N*-coordinated ruthenium nitrile complexes **3**. Unlike the reaction of cyclopropenylruthenium complexes with TMSN_3_, which yielded different products depending on the substituent at Cγ, the vinylidene complexes containing unsaturated bonds at Cδ yielded similar *N*-coordinated nitrile complexes. This transformation did not seemingly occur in the reaction of ruthenium vinylidene complexes containing Cp and PPh_3_ ligands with TMSN_3_. Deprotonation of these vinylidene complexes yielded cyclopropenyl or thermodynamic furylruthenium complexes, depending on the substitute at Cγ. Subsequent reactions of the cyclopropenyl or furylruthenium complexes with TMSN_3_ afforded different products.

## 1. Introduction

The chemical properties of metal vinylidene complexes are valuable for many organic transformations [1,2,3,4]. Vinylidene complexes of various metals also function as strategic intermediates for the catalytic conversion of alkynes [5,6], and active substrates in a series of stoichiometric reactions [7,8]. The formation of a metal vinylidene intermediate has been used to promote various carbon-carbon bond-forming reactions, with the addition of a nucleophilic carbon center to the electrophilic vinylidene α-carbon atom. This subject has been extensively reviewed [9,10]. The optimal entry into the transition metal vinylidene complexes is with the addition of electrophiles to the electron-rich carbon of metal alkynyl complexes [11,12]. Ruthenium vinylidene complexes are well-known active species in organometallic chemistry [1,5,13,14]. The vinylidene complexes of iron with dppe ligands have also been obtained [15,16,17]. The use of the acidity of the aliphatic protons on a coordinated dppe ligand in a cationic iron vinylidene complex [18] has induced the intramolecular cyclization between the dppe and vinylidene ligand. 

We believe the electron-withdrawing group at Cγ of the vinylidene complexes might play a role in the acidity enhancement of its neighboring proton. We have recently reported some preliminary results on vinylidene complexes containing indenyl and dppe ligands [19]. This study reports the synthesis and the reactivity of ruthenium vinylidene complexes containing unsaturated double bonds at Cδ, and the reactivity of these vinylidene complexes with TMSN_3_. This study also presents a deprotonation reaction of these vinylidene complexes, as well as the subsequent reactivity of the deprotonation products with TMSN_3_.

## 2. Results and Discussion

### 2.1. Preparation of Cationic Ruthenium Vinylidene Complexes ***2a**–**e***

**Scheme 1:**
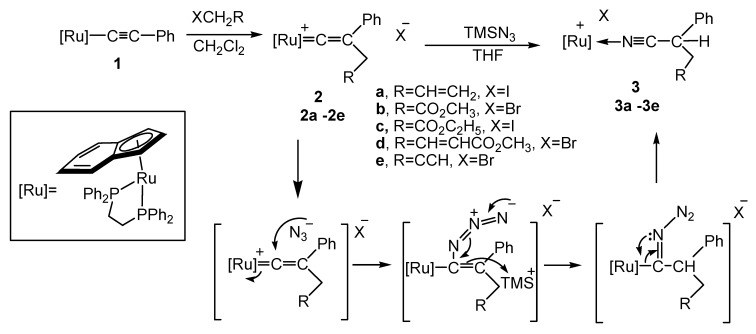
Reaction of vinylidene complexes with TMSN_3_.

Ruthenium acetylide complex **1** was prepared via deprotonation of the corresponding vinylidene precursor following the literature method [20]. The indenylruthenium vinylidene complexes **2a**–**e** containing dppe ligands and various substituents at Cγ were obtained as air-stable pink solids in 96–73% yields. The ruthenium vinylidene complexes **2a**–**e** were synthesized as shown in Scheme 1. Treatment of [Ru]–C≡C–Ph (**1**, [Ru]=(*η*^5^-C_9_H_7_)(dppe)Ru) with organic halides such as allyl iodide at room temperature produced cationic vinylidene complex **2a** in 89% yield. The ^31^P{^1^H}-NMR spectrum of **2a** exhibited a singlet at δ 76.0, indicating the chemical equivalence of the two phosphorus atoms. In the ^13^C{^1^H} NMR spectrum, the typical low field Ru–Cα resonance appeared as a triplet at δ 352.0, with a C–P coupling constant of 16.9 Hz.

**Figure 1:**
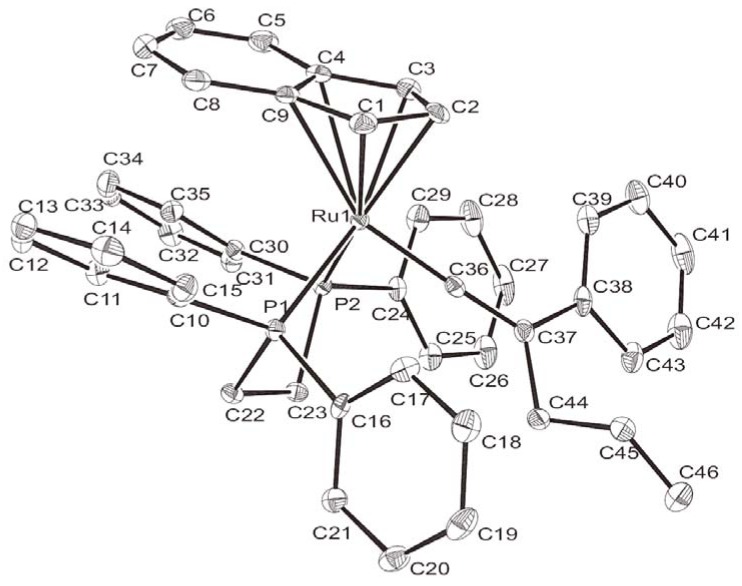
ORTEP plot of complex **2a** drawn at the 30% probability level.

Complex **2a** was air-stable at room temperature. Single crystals of **2a** suitable for X-ray diffraction analysis were obtained via recrystallization from CH_2_Cl_2_/ether. The ORTEP drawing of **2a** with thermal ellipsoids is shown at the 30% probability level in Figure 1, with selected bond distances and angles listed in Table 1. The C(37)–C(36)–Ru(1) linkage was basically linear. The C(36)–Ru(1) bond length of 1.841(4) Å indicated a typical metal-carbon bond in the vinylidene complexes [21,22]. Ruthenium vinylidene complexes containing indenyl ligand have been reported [23,24,25,26,27]. The X-ray structure of the vinylidene indenyl-ruthenium complex [Ru{=C=C(CH_3_)(C_6_H_9_)}(*η*^5^-C_9_H_7_)(PPh_3_)_2_]^+^ with bond length Ru-C(α) 1.838(5) Å [26]. The bond angle of this complex is 176.2(4) also very close linear.

Similarly, various vinylidene complexes [[Ru]=C=C(Ph)CH_2_R]^+^ **2b**–**d** (**2b**, R=CO_2_CH_3_, **2c**, R=CO_2_C_2_H_5_, **2d**, R=CH=CHCO_2_CH_3_, **2e**, R=C≡CH) were prepared using the same synthesis method as for **2a**. All indenylruthenium vinylidene complexes **2a**–**e** displayed a characteristic pink color in the solid state. These complexes were characterized via NMR spectroscopy and X-ray diffraction. In the ^1^H-NMR spectrum, the singlet or doublet resonances for the CH_2_ group at Cβ appeared at 2.2–2.8 ppm.

**Table 1:** Selected bond lengths [Å] and angles [deg] for complex **2a**.

**C(36)–Ru(1)**	1.841(4)	**C(36)–C(37)**	1.326(6)
**C(37)–C(44)**	1.538(6)	**C(44)–C(45)**	1.515(6)
**P(1)–Ru(1)**	2.3380(10)	**C(45)–C(46)**	1.321(6)
**P(2)–Ru(1)**	2.3104(10)	**P(1)–Ru(1)–P(2)**	83.29(3)
**C(37)–C(36)–Ru(1)**	177.2(4)	**C(36)–C(37)–C(44)**	119.1(4)

### 2.2. Reactivity of the Vinylidene Complexes with TMSN_3_

Cationic vinylidene complexes are known to react with alcohols or water to yield alkoxycarbene or acyl complexes, respectively [24,28,29,30,31]. The reaction is believed to proceed by nucleophilic attack at the vinylidene α carbon, followed by a proton shift from the oxonium ion to the β-carbon. A theoretical study of vinylidene complexes indicated localization of electron density on Cβ (HOMO) and the electron deficiency at Cα [32,33]. A study of the reaction of alcohols with ruthenium vinylidene complexes indicated that electron-withdrawing groups on the acetylide unit or on the metal facilitate nucleophilic attack on Cα [34]. 

The reaction of ruthenium vinylidene complex **2a** with TMSN_3_ yielded the *N*-coordinated nitrile complex **3a** as a yellow powder in 83% yield. Regardless of the equivalent ligand distribution around ruthenium, the presence of two monodentate phosphine ligands, instead of the chelating diphosphine dppe, marks a difference in the reactivity of their derivatives. Unlike the previously reported reactivity of ruthenium vinylidene complexes, the ruthenium vinylidene complex containing indenyl and dppe ligands therefore demonstrate a distinctly different reactivity from that of the Cp and PPh_3_ system.

**Figure 2:**
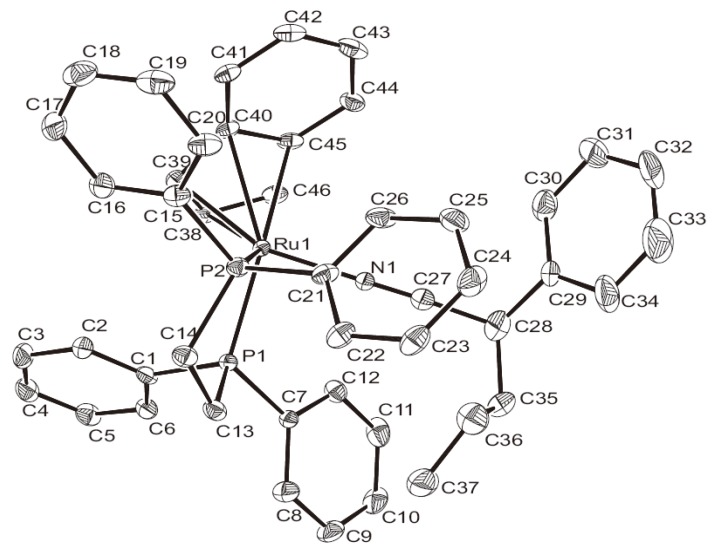
ORTEP plot of one of the stereoiomers **3a** drawn at the 30% probability level.

Complex **3a** was stable in solution and air, and soluble in polar solvents, such as CH_2_Cl_2_, acetone, and THF. Complex **3a** was characterized by ^1^H-, ^31^P-, ^13^C-NMR, as well as 2D-NMR spectroscopy. In the ^31^P{^1^H}-NMR spectrum of **3a**, two doublet resonances at δ 83.4 and 81.4 with a P–P coupling constant of 27.2 Hz indicated the presence of a enantiotopic center in the *N-*coordinated nitrile ligand. In the ^1^H-NMR spectrum, a triplet pattern at δ 3.82 with *J*_H-H_ = 7.15 Hz was assigned to the proton at this enantiotopic center. The parent peak in the HRMS spectrum of **3a** clearly indicated that **3a** resulted by adding a nitrogen atom to **2a**. Slow diffusion of the diethyl ether into a solution of **3a** in dichloromethane permitted a collection of suitable single crystals for X-ray diffraction studies (Table 2). An ORTEP diagram of one of the stereoiomers **3a** is shown in Figure 2, showing 30% thermal ellipsoids, and selected structural parameters are listed in Table 3. The nitrile ligand was coordinated to the metal center via the nitrogen atom. The bond lengths of Ru(1)–N(1) of 2.031(4) Å and N(1)–C(27) bond length of 1.130(7), respectively, were typical. The N(1)–C(27)–C(28) bond angle of 176.8° was close to 180°. X-ray analysis unequivocally confirmed the molecular structure (Table 3). 

**Table 2:** Selected bond lengths [Å] and angles [deg] for complex **3a**.

N(1)–Ru(1)	2.031(4)	C(27)–N(1)	1.130(7)
C(27)–C(28)	1.494(9)	C(28)–C(35)	1.515(10)
P(1)–Ru(1)	2.2637(13)	P(2)–Ru(1)	2.2989(15)
C(27)–N(1)–Ru(1)	174.1(6)	N(1)–C(27)–C(28)	176.8(8)
P(1)-Ru(1)-P(2)	84.34(5)	N(1)-Ru(1)-P(1)	88.88(12)
N(1)-Ru(1)-P(2)	92.02(15)		

**Table 3:** Crystal data and refinement parameters for complexes **2a** and **3a**.

	2a	3a
Empirical formula	C_46_H_41_IP_2_Ru	C_46_H_42_INP_2_Ru
Temperature	200(2) K	200(2) K
Crystal system	Orthorhombic	Monoclinic
Space group	*P n a* 2_1_	P 2_1_/n
a, Å	22.1483(8)	10.4855(3)
b, Å	11.8391(5)	19.1064(6)
c, Å	14.4101(6)	19.8563(6)
α, deg	90	90
β, deg	90	92.248(2)
γ, deg	90	90
Volume, Å^3^	3778.6(3)	3975.0(2)
Z	4	4
Crystal size, mm^3^	0.27 × 0.14 × 0.11	0.28 × 0.24 × 0.08
Refinement method	Full-matrix least-squares on F2	Full-matrix least-squares on F2
Flack parameters	−0.013(14)	
Final R indices [I > 2sigma(I)]	R1 = 0.0240, wR2 = 0.0548	R1= 0.0465, wR2 = 0.1133
R indices (all data)	R1 = 0.0283, wR2 = 0.0662	R1 = 0.0682, wR2 = 0.1318
	0.311 and −0.323 e	1.323 and −0.897 e
Largest diff. peak and hole, Å^−3^	776702	776705
CCDC number		

Conversion of a vinylidene precursor to an *N*-coordinated nitrile with hydrazine, an organometallic Beckmann rearrangement, has been reported in an iron system [35] (Scheme 1). In the Cp system, similar products can be obtained via the reaction of cyclopropenyl complex containing a phenyl group at Cγ, but the *N*-coordinated ruthenium complex product containing N_3_^−^ counter anion is unstable. An exchange of the N_3_^−^ with PF_6_^−^ stabilized the *N*-coordinated complex [36,37]. Ruthenium vinylidene complexes containing Cp and PPh_3_ ligands have been reported. However, no reaction was observed between these vinylidene complexes with TMSN_3_ [33]. In the indenyl and dppe ligands system, we previously reported that the *N*-coordinated ruthenium complex product containing N_3_^−^ counter anion is stable. This product can be obtained via the reaction of a cyclopropenyl complex with TMSN_3_ [19].

The reaction of the vinylidene precursor **2a** with TMSN_3_ may advance through the nucleophilic addition of azide anion at Cα and the electrophilic addition of a (CH_3_)_3_Si (TMS) group at Cβ. Subsequent loss of N_2_ would result in metal migration and hydrolysis of the (CH_3_)_3_Si group, to cause *N*-coordinated nitrile complex **3a**, accompanied with the halide anion (Scheme 1). In the Cp and two triphenylphosphine ligands system, no reaction occurs between vinylidene complexes and TMSN_3_, possibly due to the stereo effect of the vinylidene complexes. In the vinylidene complex **2a**, the bond angle P(1)–Ru(1)–P(2) of 83.29(3) is smaller than other vinylidene complexes containing two monodentated ligands [38,39]. The more sterically demanding two triphenylphosphine ligands compared to the bidentated dppe ligand may prevent nucleophilic addition at the Cα position of the vinylidene complexes. 

A similar reaction occurs in THF for a number of vinylidene complexes with various unsaturated substituents at Cγ, including the ester group (compounds **2b**,**c**), crotonate group (compound **2d**), and alkynyl group (compound **2e**). Reaction of vinylidene complexes **2a**–**e** with TMSN_3_ yielded similar *N*-coordinated nitrile complexes **3a**–**e** is good yield (Scheme 1). 

With the alkyl group at Cγ, vinylidene complex **2e** reacting with TMSN_3_ yielded complex **3e** as the major product, and another undetermined minor product with a 5:1 ratio. One of the well-known reactions of organic alkynes with azide can afford trizoles via cycloaddition [40]. The chemistry of the 1,3-dipolar cycloaddition of azides and alkynes is widely used in applications in organic, materials, and medicinal chemistry [41,42,43,44,45,46,47,48]. Ruthenium vinylidene complex **2e** containing an alkyl group at Cγ and reacting with TMSN_3_ yielded an *N*-coordinated complex as the major product. The electron deficiency at Cα of the ruthenium vinylidene complex **2e** may play a significant role in the reaction with TMSN_3_. That the vinyl group reacting with azide produced 1,2,3-triazoles via 3+2 cycloaddition has also been reported. Unlike the reactivity of cyclopropenyl complexes with TMSN_3_, different pathways operated depending on the substituent on the cyclopropenyl ring. The *N*-coordinated nitrile products **3a**–**e** are similar, and were air stable in solid-state and soluble in CH_2_Cl_2_, but insoluble in ether and hexane. In this series of the *N*-coordinated products, complex **3c** gave a good yield of 90%. 

### 2.3. Deprotonation Reaction of the Vinylidene Complexes

The synthesis of a number of ruthenium cyclopropenyl complexes by deprotonation of readily accessible ruthenium vinylidene complexes containing a CH_2_R group bound to Cβ has been reported [49,50,51,52,53]. A cyclopropenylruthenium complex containing pentamethylcyclopentadiethyl (Cp*) and dppp ligands was synthesized as well [50]. In the iron complexes containing Cp and dppe ligands, the vinylidene complex containing an allyl group at Cγ can be synthesized. The deprotonation reaction of this complex, the relatively more acidic proton of the dppe ligand in the cationic iron vinylidene complex could direct the reaction to proceed via a different route. The metallacyclic iron complex was obtained [54]. The intramolecular cycloaddition of two C=C bond system with an allylic ligand to produce a cyclobutylidene ring has also been reported [55]. This study focuses on the deprotonation reaction of the vinylidene complex containing an allyl group or crotonate group at Cγ.

**Scheme 2:**
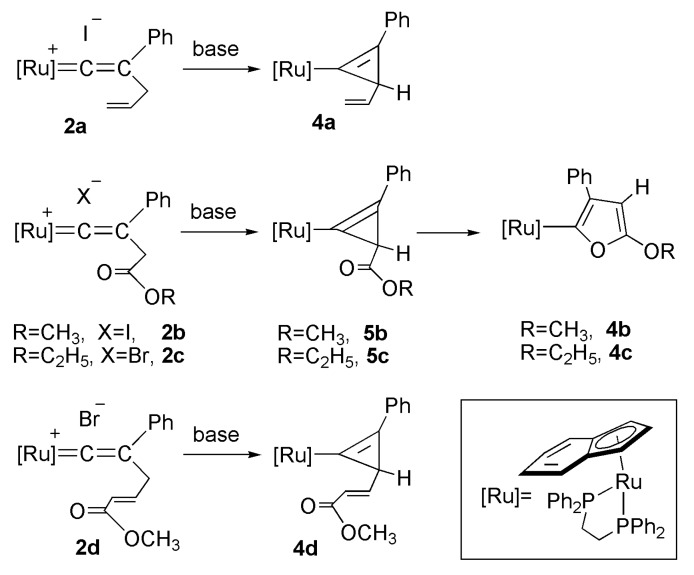
Deprotonation reaction of vinylidene complexes.

Deprotonation of the vinylidene complex **2a** via *n*-Bu_4_NOH in acetone inducing the intramolecular cyclization reaction yielded a neutral cyclopropenyl complex **4a** as a single product (Scheme 2). The reaction produces a yellow crystalline product in analytically pure form. Use of acetone or acetonitrile as a solvent produces a good yield, and use of other bases such as DBU (1,8-diazabicyclo[5,4,0] undec-7-ene) produces **4a** with a comparable yield. No metallacyclic product has been observed. The ^31^P-NMR spectrum of **4a** displays two doublet resonances at δ 94.3 and 89.3 of an AX pattern with *J*_P-P_ = 23.8 Hz, due to the presence of a stereogenic carbon center at the three-membered ring. On the ^1^H-NMR spectrum of **4a**, the methyne (methine) proton appears at δ 1.88.

The synthesis and chemical reactivity of several neutral rutheniumcyclopropenyl complexes in which the metal bonds to one sp^2^ carbon atom of the three-membered cyclopropenyl ring in the Cp system have been reported [37,56,57,58]. These cyclopropenylruthenium complexes can be prepared via deprotonation reaction of their vinylidene precursor. When a crotonate group was at Cγ, the vinylidene complex yielded a cyclopropenyl complex after deprotonation [56].

The reaction of the vinylidene complex **2b** containing an ester group at Cγ with *n*-Bu_4_NOH in acetone yields the furyl complex **4b** as the thermodynamic product (Scheme 2). The reaction proceeds via deprotonation at Cγ, followed by an intramolecular cyclization first causing the three-membered cyclopropenyl complex **5b** as the kinetic product, with a small amount of **4b** within 1h. Conversion of **5b** to **4b** is completed within 4 h. The ^31^P-NMR spectrum of **4b** displays a singlet at δ = 96.7 ppm. However, the ^31^P-NMR spectrum of the kinetic product **5b** displays a two-doublet pattern at δ = 93.8, 88.5 ppm, with *J*_P–P_ = 26.1 Hz indicating the presence of a stereogenic carbon center at the cyclopropenyl ligand. As shown in Scheme 2, the furylruthenium complex **4c** was also prepared via deprotonation of the vinylidene complex **2c** containing a ethyl acetate at Cγ. Similar reaction has been reported [52]. Deprotonation reaction of dinuclear vinylideneruthenium complexes containing an ester substituent at Cγ gave the dinuclear bisfuryl complexes [49]. Organic furan adds to [Ir(COD)(PMe_3_)_3_]Cl to yield a furyl iridium hydride complex have also been reported [59]. In the Cp and triphenylphosphine ligands system, the furyl complexes reacting with oxygen for two weeks produced the oxygen addition product [56]. In the indenyl and dppe system, complexes **4b** and **4c** are highly stable, and no oxygen addition reaction was observed.

**Scheme 3:**
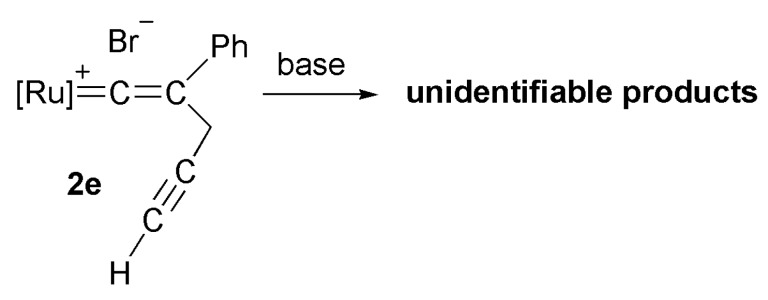
Deprotonation reaction of vinylidene complex containing alkynyl group at Cγ.

In the deprotonation reaction of the vinylidene complex containing a methyl crotonate substituent at Cγ, only the cyclopropenyl complex **4d** was obtained as the stable product. However, deprotonation of the vinylidene complex **2e** containing an alkynyl group at Cγ yielded several unidentifiable decomposition products, and no cyclopropenyl complex was observed (Scheme 3). Deprotonation of vinylidene complexes containing an ester group at Cγ produced furylruthenium complexes as thermodynamic products. 

### 2.4. Reaction of Cyclopropenyl Complexes with TMSN_3_

**Scheme 4:**
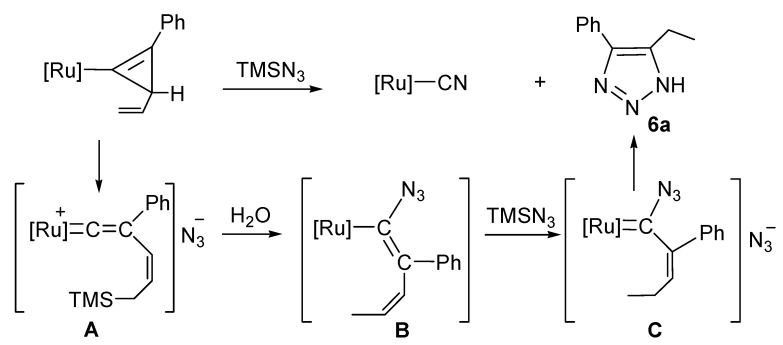
Reaction of cyclopropenyl complex containing an allyl substituent with TMSN_3_.

The reaction of the cyclopropenylruthenium complex **4a** containing an allyl substituent with an excess of TMSN_3_ resulted in the formation of a five-membered triazolate ring organic product **6a** (Scheme 4) and [Ru]–CN.

The organic product is **6a** [56]. The reaction of **4a** with TMSN_3_ results in cleavage of the C=C double bond of the cyclopropenyl ring yielding [Ru]–CN and **6a**. Scheme 4 shows a possible reaction sequence. The reaction may start with an addition of a TMS group to the double bond of the allyl group. This accompanies the opening of the three-membered ring, resulting in the formation of a cationic vinylidene intermediate **A**. Subsequent nucleophilic addition of the azide anion at Cα, accompanied with the hydrolysis of the TMS group yielded **B**. Further addition of TMS group at Cδfollowed by hydrolysis of the TMS group yielded **C**. The single- bond character of the Cα–Cβ in **B** may facilitate its cleavage. Loss of N_2_ and a [3+2] cycloaddition of the Cβ–Cγ double bond with N_3_^−^ produces the triazole **6a** and [Ru]–CN.

**Scheme 5:**
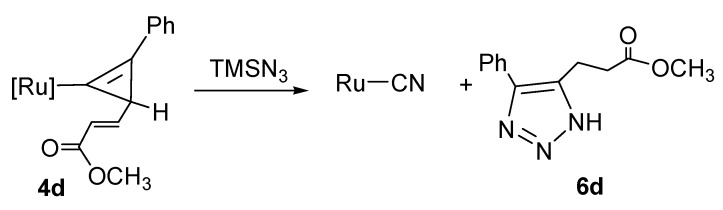
Reaction of cyclopropenyl complex containing an crotonate substituent with TMSN_3_.

We altered the substituent at Cγ to the crotonate group. The result is the same as that observed for the reaction of TMSN_3_ with the cyclopropenyl complex containing an allyl group (Scheme 5). In the reaction, the TMS group reacts with the unsaturated double bond at Cδ to induce a ring-opening reaction of the three-membered ring. No reaction occurs between the unsaturated double bond at Cδ of the vinylidene complexes and TMSN_3_. The reactivity of Cα in the vinylidene complexes causes more activity than the unsaturated bond at Cδ. 

We reported the reactivity of cyclopropenylruthenium complexes containing indenyl and dppe ligands with TMSN_3_. Various substituents at the sp^3^ carbon of the three-membered ring govern the reactivity of the cyclopropenyl complexes with TMSN_3_. The reaction of ruthenium cyclopropenyl complexes containing indenyl and dppe ligands with TMSN_3_ may proceed via an electrophilic addition of the TMS group to the three-membered ring, followed by hydrolysis to afford vinylidene intermediates containing an azide counter anion [19]. Further nucleophilic additions of N_3_^−^ at Cα, and an electrophilic addition of a second TMS group at Cβ followed by a loss of N_2_ leads to the *N*-coordinated nitrile complexes [56]. This process is similar to the chemistry of other cyclopropenylruthenium complexes. When a CN group was on the cyclopropenyl ring, a [3+2] cycloaddition of the nitrile group with azide afforded the tetrazolate complex [23]. Reactions of the cyclopropenyl complexes containing phenyl and its derivatives at Cγ yielded the stable *N*-coordinated complexes. In the Cp system, the reaction of cyclopropenyl complexes containing a methyl crotonate substituent or a vinyl substituent on the sp^3^ carbon of the cyclopropenyl ring with TMSN_3_ produced [Ru]–CN and the five-membered triazole ring. The ester group on the cyclopropenyl ring is the kinetic product, while a more stable five-membered furylruthenium product is likely the thermodynamic product. Reaction of the furylruthenium complexes with TMSN_3_ yielded [Ru]–N_3_ and the corresponding organic products by cleavage of the M–C bond. Unlike the published reactions of ruthenium cyclopropenyl complexes with TMSN_3_, which proceed through rupture of the three-membered-ring, the reaction of furylruthenium complexes with TMSN_3_ caused cleavage of the M–C bond [56]. However, those reports observed no reaction between ruthenium vinylidene complexes and TMSN_3_. 

### 2.5. Reaction of Furyl Complexes with TMSN_3_

**Scheme 6:**
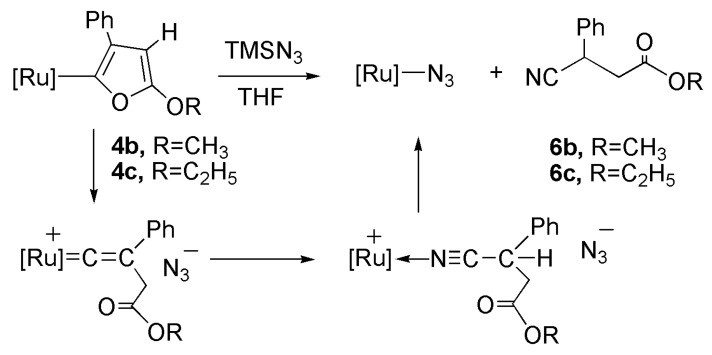
Reaction of furyl complex with TMSN_3_.

Upon applying an excess of TMSN_3_ to **4b** in THF at room temperature, the solution displayed color changes during the course of the reaction. Reaction of the furylruthenium complexes with TMSN_3_yielded [Ru]–N_3_ and the corresponding organic products by opening the five-membered ring (Scheme 6). A series of successive color changes were noted during the course of the reaction: the yellow solution of **4b** first turned red upon adding TMSN_3_ at room temperature, and subsequently turned orange after 1 h, and deep orange after 2 h. Unlike the published reactions of furylruthenium complexes with TMSN_3_, which caused cleavage of the M–C bond, the reaction of furylruthenium complexes containing indenyl and dppe ligands with TMSN_3_ were similar to the cyclopropenylruthenium complexes [56].

The X-ray diffraction analysis of the furylruthenium complex containing Cp and two PPh_3_ ligands has been reported [52]. The Ru-Cα bond length in the furylruthenium complex of 2.076(7) Å indicates a single bond. We have reported the X-ray diffraction analysis of the cyclopropenylruthenium complex containing indenyl and dppe ligands [19]. The Ru-Cα bond length in this complex is 2.028(2) Å, slightly shorter than other cyclopropenyl complexes [50,52]. This length might cause the ring-opening reaction when the cyclopropenyl or furylruthenium complexes to react with TMSN_3_. The reaction of **4b** with TMSN_3_ leading to [Ru]–N_3_ and the corresponding organic product **6b** may proceed via the similar pathway of synthesis complex **3b**. Followed by the N_3_^−^ attacks at Cα, the organic fragment can be obtained.

## 3. Experimental

### 3.1. General

All reagents were purchased from commercial sources and used without further purification. NMR spectra were obtained with a Bruker-AC 500 spectrometer at 500 MHz (^1^H), 202 MHz (^31^P), or 125 MHz (^13^C). The chemical shifts are provided in parts per million from SiMe_4_ (^1^H and ^13^C{^1^H}) or 85% H_3_PO_4_ (^31^P{^1^H}), and are reported in units of δ. Mass spectra were recorded using a LCQ Advantage (ESI) instrument. X-ray diffraction studies were conducted at the Regional Center of Analytical Instruments at the National Taiwan Normal University.

**Figure 3:**
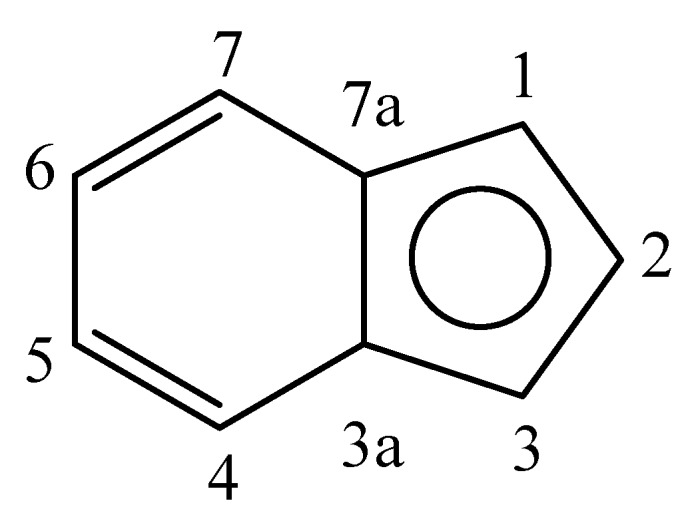
Structure of indenyl ligand.

All synthetic manipulations were performed in oven-dried glassware under nitrogen using vacuum lines and standard Schlenk techniques. Solvents were dried by standard methods and distilled under nitrogen before use. THF was distilled from sodium benzophenone ketyl and CH_2_Cl_2_ was distilled from CaH_2_. Methanol was distilled from Mg/I_2_. Complexes (η^5^-C_9_H_7_)(dppe)Ru–C≡C–Ph (**1**) [20] was prepared using the methods reported in the literature. The atom labels shown in Figure 3 were used for the ^1^H and ^13^C{^1^H} spectroscopic data:

### 3.2. Synthesis of [(η^5^-C_9_H_7_)(dppe)Ru=C=C(Ph)CH_2_CH=CH_2_][I] *(**2a**)*

To a solution of **1** (0.34 g, 0.47 mmol) in CH_2_Cl_2_ (20 mL) was added allyl iodide (0.22 mL, 2.40 mmol). After stirring overnight at room temperature, the resulting solution was concentrated to about 5 mL. The residue was then slowly added to vigorously stirred diethyl ether (40 mL). The pink precipitate thus formed was filtered off, washed with diethyl ether and hexane and dried under vacuum to give pink product **2a** (0.32 g, 0.42 mmol, 89% yield). ^1^H-NMR (CDCl_3_): δ 7.47–6.57 (m, 29H, 25H of Ph and 4H of H 4–7); 5.88 (d, 2H, H−1, 3, *J*_H−H_ = 2.7 Hz); 5.79 (t, 1H, H-2, *J*_H-H_ = 2.7 Hz); 5.24 (m, 1H, CH_2_C*H*CH_2_); 4.83, 4.63 (d, 1H each one, CH=C*H*_2_, *J*_H__−H_ = 10.1, 17.0 Hz); 2.95 (m, 4H, 2CH_2_ of dppe); 2.21 (d, 2H, *J*_H-H_ = 6.0 Hz, C*H*_2_CHCH_2_). ^31^P-NMR (CDCl_3_): δ 76.0. ^13^C-NMR (CDCl_3_): 352.0 (Cα, *J*_C-P_=16.9 Hz); 133.9–127.1 (Ph); 135.3 (*C*H=CH_2_); 126.7 (C-5, 6); 123.6 (C-4, 7); 116.8 (CH=*C*H_2_); 112.3 (Cβ); 97.1 (C-2); 79.8 (C-1, 3); 27.8 (m CH_2_ of dppe); 27.6 (*C*H_2_). HRMS (ESI, *m/z*): 757.2 (M^+^); 615.3 (M^+^-C_2_(Ph)CH_2_CH=CH_2_). Anal. Calcd. for C_46_H_41_P_2_IRu: C: 62.52, H: 4.68, Found: C: 62.71, H: 4.70.

### 3.3. Synthesis of [(η^5^-C_9_H_7_)(dppe)Ru=C=C(Ph)CH_2_CO_2_CH_3_][Br] *(**2b**)*

To a solution of **1** (0.37 g, 0.52 mmol) in CH_2_Cl_2_ (20 mL) was added methylbromoacetate (0.25 mL, 2.6 mmol). After stirring overnight at reflux temperature, the resulting solution was concentrated to about 5 mL. The residue was then slowly added to vigorously stirred diethyl ether (40 mL). The pink precipitate thus formed was filtered off, washed with diethyl ether and hexane and dried under vacuum to give pink product **2b** (0.34 g, 0.43 mmol, 83% yield). ^1^H-NMR (CDCl_3_): δ 7.48–6.86 (m, 27H, 25H of Ph group and 2H of indenyl group); 6.52 (m, 2H, H-4, 7 of indenyl group); 6.08 (m, 2H, H-1, 3 of indenyl group); 6.02 (br, 1H, H-2 of indenyl group); 3.53 (s, 3H, OCH_3_); 3.16, 2.83 (m, 2H each one, 2CH_2_ of dppe); 2.36 (s, 2H, CH_2_). ^31^P-NMR (CDCl_3_): δ 74.6. ^13^C-NMR (CDCl_3_): 350.9 (Cα, *J*_C-P_ = 16.9 Hz); 172.2 (C=O); 132.2–128.5 (Ph); 126.3 (C-5, 6); 123.6 (C-4, 7); 112.8 (Cβ); 97.3 (C-2); 80.0 (C-1, 3); 52.1 (O*C*H_3_); 29.2 (m CH_2_ of dppe); 26.8 (*C*H_2_). HRMS (ESI, *m/z*): 789.3 (M^+^); 615.2 (M^+^-C_2_(Ph)CH_2_CO_2_CH_3_). Anal. Calcd. for C_46_H_41_P_2_O_2_BrRu: C: 63.60, H: 4.76, Found: C: 64.12, H: 4.79.

### 3.4. Synthesis of [(η^5^-C_9_H_7_)(dppe)Ru=C=C(Ph)CH_2_CO_2_C_2_H_5_][I] *(**2c**)*

**1** (0.37 g, 0.52 mmol) and ethyliodoacetate (0.25 mL, 2.1 mmol) were stirred overnight at reflux in CH_2_Cl_2_ (20 mL). The purification method described for **2a** yielded pink solid product **2c** in 79% yield (0.33 g, 0.41 mmol). ^1^H-NMR (CDCl_3_): δ 7.49–6.83 (m, 27H, 25H of Ph group and 2H of indenyl group); 6.52 (m, 2H, H-4, 7 of indenyl group); 6.10 (m, 2H, H-1, 3 of indenyl group); 6.09 (br, 1H, H-2 of indenyl group); 3.95 (q, 2H, *J*_H-H_ = 7.1 Hz, OCH_2_); 3.18, 2.80 (m, 2H each one, 2CH_2_ of dppe); 2.35 (s, 2H, CH_2_); 1.14 (t, 3H, *J*_H-H_ = 7.1 Hz, CH_3_). ^31^P-NMR (CDCl_3_): δ 74.3. ^13^C-NMR (CDCl_3_): 345.4 (Cα, *J*_C-P_ = 17.2 Hz); 171.8 (C=O); 135.4–127.3 (Ph); 124.2 (C-5, 6); 122.8 (C-4, 7); 110.7 (Cβ); 98.4 (C-2); 78.2 (C-1, 3); 51.7 (O*C*H_2_); 28.8 (m CH_2_ of dppe); 25.1 (*C*H_2_); 14.7 (*C*H_3_). HRMS (ESI, *m/z*): 803.3 (M^+^); 615.2 (M^+^-C_2_(Ph)CH_2_CO_2_CH_2_CH_3_). Anal. Calcd. for C_4__7_H_4__3_P_2_O_2_IRu: C: 60.71, H: 4.66, Found: C: 60.81, H: 4.71.

### 3.5. Synthesis of [(η^5^-C_9_H_7_)(dppe)Ru=C=C(Ph)CH_2_CH=CHCO_2_CH_3_][Br] *(**2d**)*

**1** (0.21 g, 0.29 mmol) and methyl-4-bromocrotonate (0.17 mL, 1.43 mmol) were stirred overnight in CH_2_Cl_2_ (20 mL). The purification method described for **2a** yielded pink solid product **2d** in 66% yield (0.16 g, 0.19 mmol). ^1^H-NMR (CDCl_3_): δ 7.47–6.94 (m, 27 H, 25 H of Ph group and 2H of indenyl group); 6.53 (m, 2H of H-4, 7); 5.90 (m, 2H, H-1, 3); 5.74 (t, 1H, H-2); 6.32 (dt, 1H, *J*_H-H_ = 15.6; 5.5 Hz, CH_2_C*H*CH); 5.42 (d, 2H, *J*_H-H_ = 15.6 Hz, C*H*CO_2_CH_3_); 3.71 (s, 3H, OCH_3_); 3.07 (m, 4H, 2CH_2_ of dppe); 2.30 (d, 2H, *J*_H-H_ = 5.5 Hz, CH_2_). ^31^P-NMR (CDCl_3_): δ 75.9. ^13^C-NMR (CDCl_3_): 344.9 (Cα, *J*_C-P_ = 16.5 Hz); 166.5 (*C*=O); 132.3–126.8 (Ph); 135.3 (CH_2_*C*H); 126.8 (C-5, 6); 123.6 (C-4, 7); 122.1 (*C*HC=O); 112.1 (Cβ); 96.9 (C-2); 80.4 (C-1, 3); 51.5 (O*C*H_3_); 28.1 (m CH_2_ of dppe); 26.2 (*C*H_2_). HRMS (ESI, *m/z*): 815.5 (M^+^); 615.4 (M^+^-C_2_(Ph)CH_2_CH=CHCO_2_CH_3_). Anal. Calcd. for C_48_H_43_P_2_O_2_BrRu: C: 64.43, H: 4.84, Found: C: 64.81, H: 4.91.

### 3.6. Synthesis of [(η^5^-C_9_H_7_)(dppe)Ru=C=C(Ph)CH_2_C≡CH][Br] *(**2e**)*

Compound **1** (0.37 g, 0.52 mmol) and propargyl bromide (0.23 mL, 2.6 mmol) were stirred at reflux for 48 h in CH_2_Cl_2_ (20 mL). The purification method described for **2a** yielded pink solid product **2e** in 96% yield. (0.38 g, 0.50 mmol). ^1^H-NMR (CDCl_3_): δ 7.44–6.95 (m, 27H, 25 H of Ph group and 2H of indenyl group); 6.65 (m, 2H of H-4, 7); 5.95 (m, 2H, H-1, 3); 5.85 (m, 1H, H-2); 2.21 (s, 2H, CH_2_); 2.06 (s, 1H, CH). ^31^P-NMR (CDCl_3_): δ 75.4. ^13^C-NMR (CDCl_3_): 352.2 (Cα, *J*_C-P_ = 16.8 Hz); 133.8–127.2 (Ph); 125.5 (C-5, 6); 122.9 (C-4, 7); 111.7 (Cβ); 97.3 (C-2); 80.8 (C-1, 3); 83.4 (CH_2_*C*≡C); 70.3 (C≡*C*H); 28.4 (m CH_2_ of dppe); 26.9 (*C*H_2_). HRMS (ESI, *m/z*): 755.5 (M^+^); 615.4 (M^+^-C_2_(Ph)CH_2_C≡CH). Anal. Calcd. for C_46_H_39_P_2_BrRu: C: 66.19, H: 4.71, Found: C: 66.21, H: 4.73.

### 3.7. Synthesis of the N-Coordinated Complexes [(η^5^-C_9_H_7_)(dppe)Ru–NCCH(Ph)CH_2_CH=CH_2_][I] *(**3a**)*

Compound **2a** (0.11 g, 0.15 mmol) was dissolved in THF (7 mL). Next, TMSN_3_ (0.1 mL, 0.76 mmol) was added and the mixture was stirred overnight at room temperature. The resulting solution was concentrated to about 5 mL, and the residue was slowly added to vigorously stirred diethyl ether (20 mL). The yellow precipitate thus formed was filtered off, washed with diethyl ether and hexane, and dried under vacuum to give product **3a** in 83% yield (0.08 g, 0.11 mmol). ^1^H-NMR (CDCl_3_): δ 7.51–7.20 (m, 25H of Ph); 7.20, 7.07 (m, 1H each one, H of indenyl group); 6.90 (2H, H of indenyl group); 5.23 (m, 1H, CH_2_C*H*CH_2_); 5.07, 4.90, 4.86 (br, 1H each one, H of indenyl group); 4.71, 4.67 (d, 1H each one, CH=C*H*_2_, *J*_H−H_ = 10.3, 17.0 Hz); 3.82 (t, 1H, NCC*H*(Ph)CH_2_, *J*_H−H_ = 7.0 Hz); 2.48 (m, 4H, 2CH_2_of dppe); 1.95, 1.85 (m, 1H each one, CH(Ph)C*H*_2_). ^31^P-NMR (CDCl_3_): δ 83.4, 81.4 (AX, *J*_P-P_ = 27.2 Hz). ^13^C-NMR (CDCl_3_): 136.8–126.9 (Ph); 124.2 (*C*N); 118.9 (*C*H_2_=CH); 131.5 (CH_2_=*C*H); 108.2, 107.7 (C of indenyl group); 92.7 (C of indenyl group); 65.9 (C of indenyl group); 38.4 (C*C*H(Ph)); 38.3 (NCH(Ph)*C*H_2_); 28.4 (CH_2_ of dppe). HRMS (ESI, *m/z*): 772.0 (M^+^); 615.2 (M^+^-NC_2_(Ph)HCH_2_CH=CH_2_). Anal. Calcd. for C_46_H_42_P_2_INRu: C: 61.47, H: 4.71, Found: C: 61.62, H: 4.74.

### 3.8. Synthesis of the N-Coordinated Complexes [(η^5^-C_9_H_7_)(dppe)Ru–NCCH(Ph)CH_2_CO_2_CH_3_][Br] *(**3b***)

Compound **2b** (0.10 g, 0.13 mmol) and TMSN_3_ (0.1 mL, 0.76 mmol) in 7 mL of THF was stirred overnight at room temperature. The purification method described for **3a** yielded yellow solid product **3b** in 69% yield (0.07 g, 0.09 mmol). Spectroscopic data for **3b** are as follows. ^1^H-NMR (CDCl_3_): δ 7.49–7.20 (m, 25H of Ph); 7.06, 6.98 (m, 1H each one, H of indenyl group); 6.56 (2H, H of indenyl group); 4.99, 4.96, 4.90 (br, 1H each one, H of indenyl group); 4.00 (br, 1H, NCC*H*(Ph)CH_2_); 3.59 (s, 1H, OCH_3_); 2.68, 2.52 (m, 4H, 2CH_2_ of dppe); 2.28, 2.21 (m, 1H each one, CH(Ph)C*H*_2_). ^31^P-NMR (CDCl_3_): δ 83.4, 82.4 (AX, *J*_P-P_ = 27.1 Hz). ^13^C-NMR (CDCl_3_): 169.4 (CO); 133.1–126.8 (Ph); 124.3 (N*C*C); 108.1, 107.9 (C of indenyl group); 92.4 (C of indenyl group); 66.7, 66.2 (C of indenyl group); 52.2 (C*C*H(Ph)); 37.8 (O*C*H_3_); 34.4 (*C*H_2_); 28.6 (CH_2_ of dppe). HRMS (ESI, *m/z*): 804.1 (M^+^); 615.8 (M^+^-NC_2_(Ph)HCH_2_CO_2_CH_3_). Calcd. for C_46_H_42_P_2_O_2_BrNRu: C: 62.52, H: 4.79, Found: C: 62.68, H: 4.82.

### 3.9. Synthesis of the N-Coordinated Complexes [(η^5^-C_9_H_7_)(dppe)Ru–NCCH(Ph)CH_2_CO_2_C_2_H_5_][I] *(**3c**)*

Compound **2c** (0.11 g, 0.14 mmol) and TMSN_3_ (0.1 mL, 0.76 mmol) in THF (7 mL) were stirred overnight at room temperature. The purification method described for **3a** yielded yellow solid product **3c** in 64% yield (0.07 g, 0.09 mmol). ^1^H-NMR (CDCl_3_): δ 7.50–7.19 (m, 25H of Ph); 6.54, 6.53, 6.52 (m, 1H each one, H of indenyl group); 6.10 (1H, H of indenyl group); 5.02, 4.95, 4.91 (br, 1H each one, H of indenyl group); 4.04 (t, 1H, *J*_H−H_ = 5.1 Hz, NCC*H*(Ph)CH_2_); 3.98 (q, 2H, *J*_H−H_ = 7.1 Hz, OCH_2_); 2.48, 2.20 (m, 4H, 2CH_2_ of dppe); 2.18, 2.16 (m, 1H each one, CH(Ph)C*H*_2_); 1.20 (t, 3H, *J*_H−H_ = 7.1 Hz, CH_3_). ^31^P-NMR (CDCl_3_): δ 83.5, 82.2 (AX, *J*_P-P_ = 26.9 Hz). ^13^C-NMR (CDCl_3_): 171.2 (CO); 132.8–125.4 (Ph); 123.6 (N*C*C); 111.1, 109.3 (C of indenyl group); 93.7 (C of indenyl group); 67.2, 66.8 (C of indenyl group); 52.2 (C*C*H(Ph)); 42.1 (O*C*H_2_); 35.1 (*C*H_2_); 32.5 (*C*H_3_); 27.4 (CH_2_ of dppe). HRMS (ESI, *m/z*): 818.9 (M^+^); 615.4 (M^+^-NC_2_(Ph)HCH_2_CO_2_C_2_H_5_). Calcd. for C_4__7_H_4__4_P_2_O_2_INRu: C: 59.75, H: 4.69, Found: C: 59.81, H: 4.73.

### 3.10. Synthesis of the N-Coordinated Complexes [(η^5^-C_9_H_7_)(dppe)Ru–NCCH(Ph)CH_2_CH=CH CO_2_CH_3_] [Br] *(**3d**)*

Compound **2d** (0.12 g, 0.15 mmol) and TMSN_3_ (0.1 mL, 0.76 mmol) in THF (7 mL) were stirred overnight at room temperature. The purification method described for **3a** yielded yellow solid product **3d** in 60% yield (0.08 g, 0.09 mmol). ^1^H-NMR (CDCl_3_): δ 7.49–7.15 (m, 25H of Ph); 7.08, 6.92 (m, 1H, H of indenyl group); 6.55 (m, 2H, H of indenyl group); 6.39 (m, 1H, CH_2_C*H*CH); 5.3 (d, 1H, *J*_H−H_ = 15.6 Hz, CHC*H*CO); 5.08, 4.96, 4.83 (br, 1H each one, H of indenyl group); 4.27 (t, 1H, *J*_H−H_ = 6.28 Hz, NCC*H*(Ph)CH_2_); 3.77 (s, 1H, OCH_3_); 2.56, 2.16 (m, 4H, 2CH_2_ of dppe); 2.19 (m, 2H, CH(Ph)C*H*_2_). ^31^P-NMR (CDCl_3_): δ 83.9, 81.2 (AX, *J*_P-P_ = 26.8 Hz). ^13^C-NMR (CDCl_3_): 168.8 (C=O); 137.2–125.4 (Ph); 132.7 (CH_2_*C*H); 123.1 (N*C*C); 122.5 (*C*HCO); 106.9, 105.3 (C of indenyl group); 91.8 (C of indenyl group); 68.8 (C of indenyl group); 51.3 (O*C*H_3_); 38.4 (C*C*H(Ph)); 37.5 (NCH(Ph)*C*H_2_); 28.4 (CH_2_ of dppe). HRMS (ESI, *m/z*): 830.1 (M^+^); 615.5 (M^+^-NC_2_(Ph)HCH_2_CH=CHCO_2_CH_3_). Anal. Calcd. for C_48_H_44_O_2_P_2_BrNRu: C:63.37, H: 4.87, Found: C: 63.48, H: 4.89.

### 3.11. Synthesis of the N-Coordinated Complexes [(η^5^-C_9_H_7_)(dppe)Ru–NCCH(Ph)CH_2_C≡CH][Br]*(**3e**)*

Compound **2e** (0.102 g, 0.135 mmol) and TMSN_3_ (0.1 mL, 0.76 mmol) in THF (7 mL) were stirred overnight at room temperature. The purification method described for **3a** yielded yellow solid product **3e** in 66% yield (0.069 g, 0.089 mmol). ^1^H-NMR (CDCl_3_): δ 7.70–7.21 (m, 25H of Ph); 7.10, 7.07 (m, 1H each one, H of indenyl group); 6.64 (2H, H of indenyl group); 5.11, 4.98, 4.91 (br, 1H each one, H of indenyl group); 3.99 (br, 1H, NCC*H*(Ph)CH_2_); 2.74, 2.53 (m, 4H, 2CH_2_ of dppe); 2.20, (1H, C≡C*H*); 2.10, 1.99 (m, 1H each one, CH(Ph)C*H*_2_). ^31^P-NMR (CDCl_3_): δ 83.22, 82.17 (AX, *J*_P-P_ = 26.91 Hz). ^13^C-NMR (CDCl_3_): 132.5–128.1 (Ph); 124.9 (C-5, 6); 123.5 (*C*N); 121.8 (C-4, 7); 112.3 (Cβ); 97.2 (C-2); 84.1 (C-1, 3); 82.3 (CH_2_*C*≡C); 71.9 (C≡*C*H); 27.2 (m CH_2_ of dppe); 25.3 (*C*H_2_). HRMS (ESI, *m/z*): 771.4 (M^+^+1); 615.7 (M^+^-NC_2_(Ph)HCH_2_C≡CH). Calcd. for C_46_H_40_P_2_BrNRu: C: 65.02, H: 4.74, Found: C: 65.06, H: 4.77.

### 3.12. Synthesis of Cyclopropenylruthenium Complex (η^5^-C_9_H_7_)(dppe)Ru–C=C(Ph)CH－CH=CH_2_ *(**4a **)*

To a solution of **2a** (0.27 g, 0.04 mmol) in acetone (10 mL) was added a solution of *n*-Bu_4_NOH (2 mL, 2 mmol, 1M in MeOH). After the mixture was stirred at room temperature for 10 hours, the resulting solution was concentrated to about 0.5 mL. Then CH_3_CN (5 mL) was added, the yellow precipitate thus formed was filter off and washed with CH_3_CN and dried under vacuum to give the product **4a ** (0.23 g, 0.03 mmol) in 75% yield. ^1^H-NMR (C_6_D_6_): δ 7.49–6.64 (m, 29H, 25H of Ph, 4H of indenyl group); 5.70 (br, 2H, H of indenyl group); 5.41 (d, 1H, *J*_H−H_ = 16.6 Hz, H of CH=C*H*_2_); 5.24 (m, 1H, C*H*=CH_2_); 5.15 (br, 1H, H of indenyl group); 4.99 (d, 1H, *J*_H−H_ = 8.5 Hz, H of CH=C*H*_2_); 2.52, 2.34, 2.30 (m, 4H, 2CH_2_ of dppe); 1.88 (s, 1H, C*H*CH=CH_2_). ^31^P-NMR (C_6_D_6_): δ 94.3, 89.3 (AX, *J*_P-P_ = 23.8 Hz). ^13^C-NMR (C_6_D_6_): 134.3–124.7 (Ph); 128.1 (*C*H=CH_2_); 116.5 (Cα, *J*_C-P_ = 10.1 Hz); 114.3 (CH=*C*H_2_); 111.2 (C-5, 6); 106.4 (C-4, 7); 95.2 (C-2); 77.6 (C-1, 3); 26.4 (t, *J*_C-P_ = 18.4 Hz, CH_2_ of dppe); 15.8 (*C*H). HRMS (ESI, *m/z*): 756.3 (M^+^); 614.7 (M^+^-C_2_(Ph)CHCH=CH_2_). Calcd. for C_46_H_4__0_P_2_Ru: C: 73.10, H: 5.33, Found: C: 73.15, H: 5.37.

### 3.13. Synthesis of Furylruthenium Complex (η^5^-C_9_H_7_)(dppe)Ru–C=C(Ph)CH=C(OCH_3_)O *(**4b**)*

A sample of **2b** (0.25 g, 0.32 mmol) was dissolved in acetone (10 mL) at room temperature. A methanol solution of *n*-Bu_4_NOH (2 mL, 2 mmol, 1M in MeOH) was added. After the mixture stirred for 4 hours, the resulting solution was concentrated to about 0.5 mL. Then CH_3_CN (5 mL) was added the yellow precipitate thus formed was filtered off and washed with CH_3_CN. The product was dried under vacuum and identified as **4b** (0.21 g, 0.27 mmol) in 84% yield. ^1^H-NMR (C_6_D_6_): δ 7.21–6.66 (m, 29H, 25H of Ph, 4H of indenyl group); 5.43 (br, 1H, H of indenyl group); 5.15 (s, 1H, CH); 4.78 (br, 2H, H-1, 3 of indenyl group); 2.89 (s, 3H, OCH_3_); 2.73, 2.00 (m, 2H each one, 2CH_2_ of dppe). ^31^P-NMR (C_6_D_6_): δ 96.7. ^13^C-NMR (C_6_D_6_): 167.5 (CO); 140.3 (Cα, *J*_C-P_ = 16.3 Hz); 138.5–126.8 (Ph); 127.7 (=CH); 125.6 112.3 (C-5, 6); 106.2 (C-4, 7); 95.1 (C-2); 78.4 (C-1, 3); 53.1(OCH_3_); 28.5 (t, *J*_C-P_ = 19.7 Hz, CH_2_ of dppe). HRMS (ESI, *m/z*): 788.3 (M^+^); 615.4 (M^+^-C_2_(Ph)CHCO_2_CH_3_). Calcd. for C_46_H_4__0_P_2_O_2_Ru: C: 70.13, H: 5.12, Found: C: 70.21, H: 5.15. By monitoring the reaction using ^31^P-NMR spectroscopy, the kinetic cyclopropenylruthenium product **5b** was observed at the initial stage of the reaction, which gets converted to **4b** in acetone within 2 hours at room temperature. Spectroscopic data for **5b** are as follows: ^1^H-NMR (C_6_D_6_): δ 8.05–6.81 (m, 29 H, 25H of Ph, 4H of indenyl group); 6.66 (m, 2H, H of indenyl group); 6.49, 5.35, 4.86 (br, 1H, H of indenyl group); 3.65 (s, 3H, OCH_3_); 2.65, 1.84 (m, 2H each one, 2CH_2_ of dppe); 1.37 (s, 1H, CH). ^31^P-NMR (C_6_D_6_): δ 93.8, 88.5 (AX, *J*_P-P_ = 26.1 Hz).

### 3.14. Synthesis of Furylruthenium Complex (η^5^-C_9_H_7_)(dppe)Ru–C=C(Ph)CH=C(OC_2_H_5_)O *(**4c**)*

Compound **2c** (0.24 g, 0.29 mmol) and *n*-Bu_4_NOH (2 mL, 2 mmol, 1 M in MeOH) were stirred for 4 hours in acetone (10 mL) at room temperature. The purification method described for **4a** yielded yellow solid product **4c** in 83% yield (0.19 g, 0.24 mmol). ^1^H-NMR (C_6_D_6_): δ 7.48–6.66 (m, 29H, 25H of Ph, 4H of indenyl group); 5.43(br, 1H, H of indenyl group), 4.76 (s, 1H, CH); 4.78; (br, 2H, H of indenyl group), 2.89 (q, 2H, *J*_H−H_ = 7.1 Hz, OCH_2_); 2.73, 2.17 (m, 2H each one, 2CH_2_ of dppe); 0.86 (t, 3H, *J*_H−H_ = 7.1 Hz, CH_3_). ^31^P-NMR (C_6_D_6_): δ 96.4. ^13^C-NMR (C_6_D_6_): 167.5 (CO); 138.4 (Cα, *J*_C-P_ = 15.1 Hz); 136.2–127.2 (Ph); 128.5 (=CH); 124.2 117.6 (C-5, 6); 108.8 (C-4, 7); 98.7 (C-2); 80.3 (C-1, 3); 38.2 (OCH_2_); 29.4 (t, *J*_C-P_ = 18.3 Hz, CH_2_ of dppe); 15.6 (CH_3_). HRMS (ESI, *m/z*): 802.2 (M^+^); 615.3 (M^+^-C_2_(Ph)CHCO_2_C_2_H_5_). Calcd. for C_4__7_H_4__2_P_2_O_2_Ru: C: 70.40, H: 5.28, Found: C: 70.51, H: 5.31. By monitoring the reaction using ^31^P-NMR spectroscopy, kinetic cyclopropenylruthenium product **5c** was observed at the initial stage of the reaction which gets converted to **4c** in acetone within 2 hours at room temperature. Spectroscopic data for **5c** are as follows: ^1^H-NMR (C_6_D_6_): δ 7.84–6.53 (m, 27H, 25H of Ph, 2H of indenyl group); 6.52 (m, 2H, H of indenyl group); 6.34, 5.72, 5.11 (br, 1H, H of indenyl group); 3.81(q, 2H, *J*_H−H_ = 6.8 Hz, OCH_2_); 2.87, 1.93 (m, 2H each one, 2CH_2_ of dppe); 1.24 (s, 1H, CH); 1.05 (t, 3H, *J*_H−H_ = 6.8 Hz, CH_3_). ^31^P-NMR (C_6_D_6_): δ 92.4, 89.1 (AX, *J*_P-P_ = 24.3 Hz).

### 3.15. Synthesis of Cyclopropenylruthenium Complex (η^5^-C_9_H_7_)(dppe)Ru–C=C(Ph)CHCH=CHC(O) OCH_3_ *(**4d**)*

Compound **2d** (0.24 g, 0.29 mmol) and *n*-Bu_4_NOH (2 mL, 2 mmol, 1 M in MeOH) were stirred for 10 hours in acetone (10 mL) at room temperature. The purification method described for **4a** yielded yellow solid product **4d** in 79% yield (0.19 g, 0.23 mmol). ^1^H-NMR (C_6_D_6_): δ 7.38–6.85 (m, 28H, 25H of Ph, 2H of indenyl group, 1H of C*H*C=O); 6.48 (br, 2H, H of indenyl group); 6.35 (m, 1H, C*H*=CHC(O)); 5.43, 5.35, 5.01 (br, 1H each one, H of indenyl group); 3.58 (s, 1H, OCH_3_); 2.47, 2.41, 1.85 (m, 4H, 2CH_2_ of dppe); 1.30 (s, 1H, C*H*CH=CHC(O)). ^31^P-NMR (C_6_D_6_): δ 92.7, 89.9 (AX, *J*_P-P_ = 24.7 Hz). ^13^C-NMR (CDCl_3_): 171.3 (=CH); 169.2 (CO); 133.1–128.7 (Ph); 117.4 (=CH); 111.8 (C-5, 6); 107.4 (C-4, 7); 96.3 (C-2); 76.9 (C-1, 3); 55.2 (OCH_3_); 34.3 (*C*H); 28.4 (t, *J*_C-P_ = 19.7 Hz, CH_2_ of dppe). HRMS (ESI, *m/z*): 814.1 (M^+^); 615.2 (M^+^-C_2_(Ph)CHCHCO_2_CH_3_). Calcd. for C_4__8_H_42_P_2_O_2_Ru: C: 70.84, H: 5.20, Found: C: 70.96, H: 5.24.

### 3.16. Reaction of ***4a*** with TMSN_3_

To a solution of **4a** (0.15 g, 0.20 mmol) in THF (5 mL) was added TMSN_3_ (0.1 mL, 0.76 mmol). The solution was stirred overnight at room temperature. Then the solvent was reduced under to about 2 mL, and slowly added to stirring hexane (20 mL). The orange precipitates thus formed were filtered off and wash with hexane and identified as [Ru]-CN (0.08 g, 0.12 mmol) in 60% yield. The organic product was extracted with hexane and collected by extraction with hexane and purified by chromatography, then, the solvent was removed under vacuum to give **6a** (0.015 g, 0.087 mmol, 44% yield). Spectroscopic data for **6a** are as follows [37]. ^1^H-NMR (CDCl_3_): δ 7.35–7.12 (m, 5H, H of Ph group); 2.75 (q, 2H, CH_2_, *J*_H−H_ = 7.3 Hz); 1.31 (t, 3H, CH_3_, *J*_H−H_ = 7.3 Hz). High resolution MS: 173.1(M^+^). Calcd. for C_10_H_11_N_3_: C: 69.34, H: 6.40, Found: C: 69.36, H: 6.41. Spectroscopic data for **[Ru]**–**CN** are as follows: ^1^H-NMR (CDCl_3_): δ 7.40–7.01 (m, 22H, 20H of Ph group and 2H of indenyl group); 6.99 (m, 2H, H-4,7 of indenyl group); 5.18 (br, 1H, H-2 of indenyl group); 4.97 (d, 2H, *J*_H−H_ = 2.1 Hz, H-1,3 of indenyl group); 2.55, 2.28 (m, 2H each one, CH_2_ of dppe). ^31^P-NMR (CDCl_3_): δ 86.2.

### 3.17. Reaction of ***4b*** with TMSN_3_

Compound **4b** (0.15 g, 0.19 mmol) and in TMSN_3_ (0.1 mL, 0.76 mmol) were stirred overnight at room temperature in THF (5 mL). The purification method described for **6a** yielded an orange precipitate of [Ru]–N_3_ (0.10 g, 0.15 mmol) in 79% yield and the organic product **6b** (0.02 g, 0.11 mmol) in 58% yield. Spectroscopic data for **6b** are as follows: ^1^H-NMR (CDCl_3_): δ 7.25–7.11 (m, 5H, H of Ph group); 3.85 (dd, 1H, C*H*(Ph), *J*_H−H_ = 6.2, 7.9 Hz); 3.32 (s, 3H, OCH_3_); 2.52, 2.31 (AB, 2H, CH_2_, *J*_H−H_ = 7.9, 16.3 Hz and *J*_H−H_ = 6.2, 16.3 Hz). ^13^C-NMR (CDCl_3_): δ 171.2 (CO_2_); 132.1–128.4 (Ph); 120.2 (CN); 52.4 (OCH_3_); 38.8 (CH_2_); 31.3 (CH). HRMS: 189.2 (M^+^). Calcd. for C_11_H_11_O_2_N: C: 69.83, H: 5.86, Found: C: 69.88, H: 5.87.

### 3.18. Reaction of ***4c*** with TMSN_3_

Compound **4c** (0.18 g, 0.20 mmol) and in TMSN_3_ (0.1 mL, 0.76 mmol) were stirred overnight at room temperature in THF (5 mL). The purification method described for **6a** yielded an orange precipitate of [Ru]–N_3_ (0.08 g, 0.12 mmol) in 60% yield and the organic product **6c** (0.02 g, 0.09 mmol) in 45% yield. Spectroscopic data for **6c** are as follows: ^1^H-NMR (CDCl_3_): δ 7.32–7.24 (m, 5H, H of Ph group); 4.12 (dd, 1H, C*H*(Ph), *J*_H−H_ = 7.2, 8.1 Hz); 3.57 (q, 2H, OCH_2_, *J*_H−H_ = 7.8 Hz); 2.58, 2.46 (AB, 2H, CH_2_, *J*_H−H_ = 8.1, 16.6 Hz and *J*_H−H_ = 7.2, 16.6 Hz); 1.3 (t, 3H, CH_3_, *J*_H−H_ = 7.8 Hz). ^13^C-NMR (CDCl_3_): δ 169.4 (CO_2_); 134.3–125.1 (Ph); 118.1 (CN); 51.7 (OCH_2_); 39.4 (CH_2_); 33.7 (CH); 15.4 (CH_3_). HRMS: 203.1 (M^+^). Calcd. for C_12_H_13_O_2_N: C: 70.92, H: 6.45, Found: C: 70.94, H: 6.46.

### 3.19. Reaction of ***4d*** with TMSN_3_

Compound **4d** (0.15 g, 0.18 mmol) and in TMSN_3_ (0.1 mL, 0.76 mmol) were stirred overnight at room temperature in THF (5 mL). The purification method described for **6a** yielded an orange precipitate of [Ru]–CN (0.07 g, 0.11 mmol) in 61% yield and the organic product **6d** (0.017 g, 0.074 mmol) in 41% yield. Spectroscopic data for **6d** are as follows [52]: ^1^H-NMR (CDCl_3_): δ 7.42–7.23 (m, 5H, H of Ph group); 3.51 (s, 3H, OCH_3_); 3.11 (t, 2H, CH_2_, *J*_H−H_ = 7.2 Hz); 2.74 (t, 2H, CH_2_, *J*_H−H_ = 7.2 Hz). HRMS: 231.1(M^+^). Calcd. for C_12_H_13_O_2_N_3_: C: 62.33, H: 5.67, Found: C: 62.34, H: 5.68. 

### 3.20. X-ray Analysis of ***2a*** and ***3a***

Crystal data and refinement parameters for complexes **2a** and **3a** are listed in Table 3. CCDC-776702, 776705 (see Table 3) contain the supplementary crystallographic data for this paper. These data can be obtained free of charge from The Cambridge Crystallographic Data Centre via http://www.ccdc.cam.ac.uk/data_request/cif.

## 4. Conclusions

Ruthenium vinylidene complexes containing indenyl and dppe ligands and unsaturated bonds at Cδ can be synthesized in good yield. Reaction of these vinylidene complexes with TMSN_3_ yielded *N*-coordinated complexes as the stable products. Deprotonation of vinylidene complexes containing allyl or crotonate group at Cγ yielded cyclopropenylruthenium complexes as a single product. When an ester group is at Cγ, furylruthenium complexes can be obtained as the thermodynamic products. The corresponding kinetic cyclopropenylruthenium products can be observed in the initial stage. Reaction of the cyclopropenylruthenium complexes with TMSN_3_ yielded [Ru]–CN, and the corresponding organic compounds via transformation of the vinyl group to an ethyl group. Reaction of the furylruthenium complexes with TMSN_3_ yielded [Ru]–N_3,_ and the corresponding organic compounds via opening the five-membered ring to form the *N*-coordinated intermediate. 
